# Hunting the Cell Cycle *Snark*

**DOI:** 10.3390/life14101213

**Published:** 2024-09-24

**Authors:** Vic Norris

**Affiliations:** Laboratory of Bacterial Communication and Anti-Infection Strategies, EA 4312, University of Rouen, 76000 Rouen, France; victor.norris@univ-rouen.fr

**Keywords:** cell division, bacteria, hyperstructure, water, phase separation, condensate, origins of life, chromosome replication, phenotypic diversity, differentiation

## Abstract

In this very personal hunt for the meaning of the bacterial cell cycle, the *snark*, I briefly revisit and update some of the mechanisms we and many others have proposed to regulate the bacterial cell cycle. These mechanisms, which include the dynamics of calcium, membranes, hyperstructures, and networks, are based on physical and physico-chemical concepts such as ion condensation, phase transition, crowding, liquid crystal immiscibility, collective vibrational modes, reptation, and water availability. I draw on ideas from subjects such as the ‘prebiotic ecology’ and phenotypic diversity to help with the hunt. Given the fundamental nature of the *snark*, I would expect that its capture would make sense of other parts of biology. The route, therefore, followed by the hunt has involved trying to answer questions like “why do cells replicate their DNA?”, “why is DNA replication semi-conservative?”, “why is DNA a double helix?”, “why do cells divide?”, “is cell division a spandrel?”, and “how are catabolism and anabolism balanced?”. Here, I propose some relatively unexplored, experimental approaches to testing *snark*-related hypotheses and, finally, I propose some possibly original ideas about DNA packing, about phase separations, and about computing with populations of virtual bacteria.

## 1. Introduction

In responding to the invitation from this journal to relate my scientific background, I try to avoid this being a purely narcissistic trip by casting it as a hunt in which the nature of the hunt and the quarry is what matters rather than the hunter. Lewis Carroll’s *The Hunting of the Snark* is a poem that is open to many interpretations, one of which is that it “is an allegory for the search for happiness” [1], whilst parts of it are “attempts to create a sense of order and meaning out of chaos” [2]. In this poem, the nature of the hunt is unclear; indeed, the nature of the quarry itself, the *snark*, is unclear. It may even be dangerous. In replying to the invitation from this journal to recount my scientific experiences, the *Hunting of the Snark* resonates. My interdisciplinary adventures find an echo in the diversity of the members of the hunt for the *snark* whilst some of my companions in these adventures have vanished, again, like a member of the hunt. Personal relationships help determine the success of a scientific project as they do in the hunt where the Butcher reveals that “he could only kill beavers”, which is not good news for the Beaver, another member of the hunt. Having or not having access to the right equipment is also important, which resonates with “He had forty-two boxes, all carefully packed … They were all left behind on the beach”. Classing the *Hunting of the Snark* as a nonsense poem is not to be disparaging: exploring nonsense is a serious, fertile business that creates novel insights, as can happen in science. Here, the quarry is the nature of the system regulating the bacterial cell cycle. I describe how my collaborators and I have hunted it in the places I have worked and how our ideas about it have changed.

## 2. Orsay, France 1981–1983

My scientific career began in France. I had a motley collection of qualifications and experience in literature, psychology, computer science, and mathematics and was struggling to find a way to enter the research world. Going ‘door-to-door’ on the campus in Orsay, I eventually met Pierre Schaeffer, the head of the *Institut de Microbiologie*, who, along with his colleague Jean Brevet, kindly allowed me to work as a trainee technician in Jean’s group. Pierre himself and his colleagues had carried out some very original work about possible states of diploidy in *Bacillus subtilis* following the fusion of protoplasts [3]; similar work on epigenetics was being carried out by Luisa Hirschbein and her collaborators in the same institute [4] (I was to return to the subject of phenotypic diversity much later). These collaborators included Françoise Lehegarat, who found that a ribosomal protein bound DNA [5], an example of multi-tasking, which I only appreciated later. Other work that I was to appreciate later in the context of phenotypic diversity was the work on the pausing of DNA replication performed by Simone Seror and her lab [6].

In Jean Brevet’s group, I worked with Françoise Gosti-Testu, Dhia Hassan, Mireille Larribe, and Denise Borowski on the transposition of Tn7 in *E. coli*. I tried to show that the reason transposons were not found in the chromosomes of all the bacteria in a species was because they incurred a metabolic cost that would disadvantage them with respect to their competitors. This led to my obtaining a French research qualification that, coupled with the support from my friends in Orsay like Celine Karmazyn-Campelli, opened the door to my continuing in research by joining Dick D’Ari’s lab in the *Institut Jacques Monod* in Paris.

## 3. Institut Jacques Monod, France 1983–1986

The subject of my PhD was the nature of the coupling between chromosome replication and subsequent cell division in *E. coli*. Interrupting this replication prevents division, which leads to filamentation [7]. This is not only due to the induction of the SOS response but also of a second mechanism [7,8]. Dick entertained the nice idea that the replication of a specific sequence in the terminus was required—a termination function—and that if this replication were prevented, cells would not divide. He, therefore, thought that temperature-sensitive mutants in which this function would be defective might exist in the Hirota collection of mutants held in the *Institut Pasteur* [9]. My task was to investigate a couple of them using temperature shifts and pulse-labeling. Neither turned out to encode a termination function, although, ironically, one of them was mutated in the primase, DnaG (part of the intriguing *MMS* operon [10]), and was affected in replication, but in the initiation step. After transfer to the non-permissive temperature, this mutant first filamented and then began to produce anucleate cells [11]. As a control, I subjected the parental strain to the same temperature shift and was disconcerted to find that the cells first filamented and then divided to become smaller than previously. Willie Donachie, who was visiting Dick’s lab, remarked that the results of the controls were often more interesting than those of the experiments themselves. In parallel with this, Aline Jaffé in the lab was studying anucleate cell production by cells in which chromosome replication was perturbed and, in particular, how this production depended on cAMP [12]. I was to return to these matters many years later.

Dick’s lab was a great place to learn about the fundamentals of biology. I learnt about the concept of the ‘initiation mass’, the cellular mass per origin of replication at which chromosome replication is initiated. This was an idea of Willie Donachie’s [13] that was shared by Bob Pritchard and his collaborators [14]. I also learnt about the *Replicon Theory*, according to which a replicon consists of a replicator (a DNA sequence) and a structural gene for an initiator protein [15,16] that was later identified as DnaA [17]. Arthur Koch was another visitor to the lab; Arthur gave unforgettable, interdisciplinary talks in which he would blow soap bubbles to show the relevance of their physics to cell division or cut pairs of tights with scissors to show the importance of surface stress to the dynamics of peptidoglycan [18]. Larry Rothfield was also a visitor to the institute; he was working on what he termed ‘periseptal annuli’ [19] and ‘nucleoid occlusion’ [20,21]. Later, in one of those ironies that seem to structure our existence (see below), he was to identify RNaseE as a cytoskeletal element of *E. coli* [22]. The cell cycle hypothesis I found the most satisfying was Neil Mendelson’s ‘helix clock’, which was based on the insertion of peptidoglycan (needed for growth), necessarily leading to the opening of strands of the chromosome that was attached to the peptidoglycan [23]; the helix clock made a lot of sense as an integrative model that brought together the essential characteristics of bacterial cells. Dick himself wanted to find out what information *E. coli* used in order to couple cell cycle to the growth rate; this entailed him performing experiments in which he varied the combinations of certain amino acids in the growth media. As I was proving during my thesis that I was not a gift to experimental science, we reversed roles and I had a lot of fun discussing his results with him (he continued successfully with this story, as in [24]).

The *Institut Jacques Monod* had many interesting people with whom I was able to interact. The group of Adam Képès had developed an intriguing way to synchronize populations of *E. coli* based on cycles of phosphate limitation [25]; François Képès in Adam’s group helped me greatly with experiments, including those requiring the Coulter Counter, a machine that I was using to obtain the distributions of bacterial cell sizes but that was prone to having its orifice irrecoverably blocked (for me) at inconvenient times. Claude Reiss and Jaime Gabarro-Arpa had a program for predicting the probability of Z-DNA forming from a DNA sequence [26]. Hoping that DNA supercoiling might increase with growth until a threshold at which Z-DNA would form as the signal to trigger replication, I laboriously typed in the sequence of the origin region of *E. coli*, where their program did seem to suggest that Z-DNA might form. Masamichi Kohiyama’s group was working on the initiation of chromosome replication in *E. coli* (Michi himself had conducted the pioneering work needed to identify DnaA as the key initiator protein [17,27,28]) and I learnt about in vitro replication systems from my interactions with Patrick Hughes and John Herrick in his group [29,30]. A couple of other dynamic groups, those of Dusko Ehrlich and Miro Radman, also worked on bacteria and DNA replication [31,32], and I benefitted from many fruitful exchanges with them. Jacques Ninio in the institute impressed me with his insights into a wide range of subjects (for example, [33,34,35]) and by showing me how to improve a program I had helped write in Orsay to search for similarities in DNA sequences: Jacques sped it up by over two orders of magnitude. Jacques also helped with a learning program, *Coco*, I had started writing back in the 1970s. *Coco* was originally intended to model a hypothesis about how brains work, the idea being that the process of thinking requires us to reconcile having thoughts that are coherent with the present environment with having thoughts that are coherent with one another over time (see below) [36].

Aline Jaffé helped me investigate the idea that the placing of the division site is governed by macromolecules moving in an electric field. Aline looked, somewhat uneasily I have to say, through a microscope whilst I fumbled to connect electrodes to either end of a bacteria-containing slide. I therefore devised a better system. This comprised a Petri dish with a postage stamp-sized electrode under the nutrient agar, a filter with bacteria in different orientations, a piece of insulating parafilm, and a needle as the other electrode. The electrodes were connected to a powerful generator that allowed me to impose a gradient of around 100,000 V cm^−1^. I had little success in showing that I could displace the septum even though I did once produce short filaments (but which I reluctantly attributed to the effects of a heat shock [37]). Then, one day, I pierced the parafilm with the needle: there was a blue flash, a loud bang, and the needle was vaporized; this spelt the end to these experiments (but see below).

It was clear to Dick and Aline that the bacterial cell cycle was not governed by the zonal growth of the envelope as proposed as part of the influential *Replicon Theory* [15,38]. In this critical environment and with little personal investment in the then-current paradigms, I began to wonder whether the prokaryotic and eukaryotic cell cycles might be regulated in essentially the same way. Françoise Gosti-Testu (see above) told me how important calcium fluxes were in the cell cycle of eukaryotic cells, while Barry Holland, on a visit to Dick’s lab, told me that Eli Orr in his department in Leicester had found a myosin in yeast [39]. It was not a big step to imagine that bacteria had a cytoskeleton and that the bacterial cell cycle was regulated by calcium. Barry was starting the ‘reverse genetics’ approach of going from identification in *E. coli* of a candidate eukaryotic-like protein to studying the phenotype of the corresponding mutant. To join Barry’s exciting project, I needed funding. I therefore applied for a long-term EMBO fellowship and was interviewed by Kurt Nordstrom in Uppsala. At the end of the interview, I asked Kurt about his own research. He told me how he was studying minichromosome replication in a strain in which the host chromosome was itself replicated at random from a plasmid origin (‘integrative suppression’) rather than from its normal origin (*oriC*). I failed to realize that this was to be the basis of what should be considered one of biology’s most fundamental experiments [40].

## 4. Leicester, UK 1986–1996

As a postdoc in Barry’s lab in the Department of Genetics, I collaborated with another postdoc, Serge Casaregola, to try to clone a myosin-encoding gene from *E. coli*. The classical eukaryotic myosins have a high MW and my approach entailed using very long, gradient gels to purify two very high MW proteins from *E. coli*. The polyclonal antibodies raised to these proteins were then used by Serge to clone a gene that, sadly, encoded not a myosin but RNaseE [41]. This was a disappointment, but worse was to follow. We then learnt that Sota Hiraga’s group had obtained a mutant that affected chromosome segregation and that the gene responsible, *mukB*, encoded a high MW protein [42]. It corresponded of course to our other protein. Barry was very open to scientific discussion and speculation, and in parallel with our search for myosin, Mao Chen, Jan Voskuil, Gordon McGurk, and I began a search in Barry’s lab for the elements of a system that could allow calcium to regulate the cell cycle of *E. coli* and its putative cytoskeleton [43]. This entailed looking for a calmodulin (we were encouraged by the discovery of this protein in another bacterial species [44]) and for a voltage-operated calcium channel. We did this using drugs that inhibit the eukaryotic versions of these proteins, like compound 48/80 and nifendipine, to which *E. coli* is sensitive, with the hope of obtaining mutants affected in calcium signaling and the cell cycle [45,46]; it is worth noting that the sensitivity of bacteria to these type of drugs has implications for the gut microbiome [47]. One idea we found attractive was that calcium served as a reset signal [48]. Although we made some progress, the calcium story in Leicester ended when Barry left to go to Orsay, where he continued to advance it with Simone Seror and Tony Campbell (one intriguing finding was that an extracellular concentration of Ca^2+^ of ten millimolar raised ATP levels in *E. coli* by 30% and lowered its generation time by 10% [49]). The calcium channel story also ended for me because I had learnt from Arthur Kornberg about the work of Rosetta Reusch on non-proteinaceous ion channels, on a major class of post-translational modifications, and several other fundamental subjects, all of which have been largely ignored by the scientific community [50,51,52,53,54,55,56]; in particular, Rosetta and her collaborators showed that the combination of poly-(R)-3-hydroxybutyrate and polyphosphate could form voltage-gated calcium channels in membranes [57]. Given the universality of these simple molecules (see below), I concluded that *E. coli* had no need for a proteinaceous channel. I therefore turned my attention to looking in *E. coli* for another eukaryotic signal transduction pathway, this time one that depended on protein kinase C (PKC). This choice was because Eli Orr, whose lab was in the same department, was looking for a PKC in yeast (as an aside, Eli, in the pursuit of his own *snark*, discovered a way to make antibodies specific to different cancers but died before he could exploit it). I learnt that some isozymes of PKC are stimulated by calcium and by phospholipids to phosphorylate their cytoskeletal targets on serine, threonine, and tyrosine residues [58]. Eli kindly provided advice and materials; then, helped by Sean Sweeney, by Tom Baldwin in Peter Williams’ lab, and by Karen Leach in Kalamazoo, we obtained some indirect evidence for the existence of a PKC in *E. coli* [59] but were never able to clone a gene encoding a genuine PKC.

I moved from the Department of Genetics to the Department of Microbiology and Immunology, where I managed to obtain funding to set up my own group (Primrose Freestone, Mirella Trinei, Istvan Toth, Susan Grant, James Canvin, and Kishor Modha) to look in *E. coli* for eukaryotic-like protein kinases, which phosphorylate proteins on serine, threonine, and tyrosine residues (STY). Though the existence of STY kinases in bacteria had already been demonstrated by others [60], the paradigm remained that bacteria only had kinases that phosphorylated proteins on histidine residues. We used both a pathogenic strain of *E. coli* and an L-form of *E. coli*. In the latter case, we collaborated with Tetsuo Onoda and Akinobu Oshima, who were generous with their materials and results [61]; hoping that cytoskeletal elements would be upregulated in their L-forms, we determined the level of FtsZ, but it turned out to be lower than in the parental strain [62] (it can in fact be deleted [63]), while the most abundant protein was YfiD, which encodes a stress-induced subunit of pyruvate formate lyase [61]. We did manage to identify some proteins phosphorylated on serine, threonine, and tyrosine like the Universal Stress Protein, UspA, and ribosomal protein S1 in an enteropathogenic strain of *E. coli* [64,65], along with a protein that interacts with the translational machinery, TypA (or BipA), in both this pathogenic strain and in an L-form of *E. coli* [61,66]. Unfortunately, none of these proteins seemed to offer a clear route to working on the cell cycle, on which I continued to reflect.

Calcium is usually at a much higher concentration outside a cell than within it. Hence, major compositional or structural changes to the bacterial membrane itself might generate a calcium flux. In reading about bilayers, I learnt that, in vitro at least, the transfer of a phospholipid from one monolayer to the other is extremely slow. One evening, as I was running past the University cricket grounds, I experienced a ‘divine revelation’: the packing pressure in the inner monolayer increases as a bacterium grows until it reaches a threshold at which the integrity of the bilayer is disrupted and there is a massive transfer of phospholipids from the inner to the outer monolayer (which resets the membrane clock). This transfer or *flip-out* would generate a calcium flux and would constitute the major cell cycle signal [67]. I was particularly happy about this hypothesis because it grounded the nature of the regulation of the cell cycle of modern cells in the solution to a fundamental problem that may have confronted early cells. Although I was later to doubt this hypothesis (and to wonder about ‘divine revelations’), I remained attached to the idea that the solution to problems confronting modern cells might be found in the solutions to problems confronting early cells.

In reading about the transverse asymmetry of membranes, I could not avoid reading about their lateral asymmetry. The evidence for membrane domains combined with the ideas of Itzhak Fishov, who spent some time as a visitor to my lab, and of Conrad Woldringh, led me to speculate that the interaction of the chromosome with the membrane via the coupled transcription–translation–insertion of proteins (which I called *transertion*) might not only structure the membrane but also facilitate chromosome segregation and the positioning of the division site [68] (these ideas about transverse, lateral asymmetry, and division had testable implications for phospholipid translocases [69]).

I have long been attracted to speculating, which could be justified by likening science to a hunt. For such hunts to succeed, the hunters need to be complementary and adopt the strategy of Lévy flights (random walks with the probability of a given step-length being inversely proportional to its length) with the theoretical biologist providing the big, speculative jumps. A chance encounter in the University canteen with Mark Madsen, a mathematician, resulted in our setting up a theoretical biology group. One of our publications was based on membrane domains and what I saw as the inevitability of differentiation during the cell cycle [70]; this is because two or more copies of the same gene are present in the same cytoplasm during chromosome replication and, with a global negative regulation *in trans* and a local positive regulation *in cis*, one copy will be expressed at the expense of the other copy (much later, I realized that this spontaneous differentiation via the cell cycle might be an example of a *spandrel* [71]). Another idea related to membrane domains was that the sequestration of newly replicated origins of replication involved a lipid compartment [72]. In this context of membranes and the cell cycle, I began to wonder why cells bothered initiating the replication of their DNA at one particular mass (or origin to mass ratio) rather than another. One possibility is that cells are under a selective pressure to replicate their DNA before transcription becomes limiting for growth and their solution is to sense the approaching limit via a ‘transcriptional sensing’ in the form of a phase transition in the membrane occurring at a critical density of *transertion* [73]. Unfortunately, this idea seemed to me at the time to be incompatible with the finding of Eliasson and Nordstrom that the signal for initiation was given on time even when the replication of the chromosome itself was being initiated at random [40].

Martin Goldberg in Barry’s lab introduced me to an engineer, Colin Gibson, who was looking for a biologist with whom he might collaborate. Colin was interested in the esoteric world of giant dipole oscillations that, it was speculated, might play an important role in biological systems and that could be influenced by exposure to low intensity, non-ionizing radiation in the GHz and THz frequencies [74,75,76,77]. Such coherent collective vibrational modes could, conceivably, drive the cell cycle, and the pursuit of this idea led to my collaborating with physicists in the *University of London* (like Glenn White and Derek Martin), the *University of Warwick* (Frohlich’s former student, Gerard Hyland), and engineers at *British Aerospace* (like Norman Grant). Gerard and I tried to use this idea to explain the intriguing results of a series of bold experiments conducted by Michio Matsuhashi and his collaborators on sonic communication in bacteria [78,79].

In order to grow, *E. coli* must insert new strands of peptidoglycan into its wall. This entails breaking existing links between the strands, which puts the bacterium at risk of lysing given the pressure across the wall. Taking a leaf out of Arthur Koch’s book, Bill Manners in the Engineering department and I proposed the *Hernia model* to explain how the envelope could be preferentially strengthened where it is weakest via the effects on the peptidoglycan metabolism of the deformation of the cytoplasmic membrane [80]. One day I received a paper out of the blue from Ann Rajnicek showing how bacteria growing on a surface altered their walls and bent in response to a weak electric field of only a few volts per cm [81]; some of these bacteria resembled horseshoes with their DNA migrating into the poles. This *déjà vu* led to Ann and I submitting a grant to combine this “in vivo electrophoresis” with immunochemistry so as to perform a bacterial vivisection and provide information about bacterial structures. Our application was, however, rejected.

To advance our theoretical biology group, Mark and I collaborated with Shaun Heaphy, a virologist, and Dave Snelling, a computer scientist, to model the value of trying to treat viral diseases with engineered versions of *defective interfering particles* [82,83]. We were unable to publish our model in one of the ‘upmarket’ journals; we needed such endorsement to obtain the funds to develop the model so we could take it no further (that said, I did return to it much later in the context of COVID-19 [84,85]). Hypotheses were something I discussed with Mick Pocklington in Eli’s group. Mick was a font of knowledge and would give me updates on the recent literature when we had lunch together in the University canteen. Mick himself had some original ideas about the connectivity of biological systems, a subject I was to work on myself later (see below).

All this activity fitted well with the interests of our theoretical biology group but less well with those of our experimental group, which, because of the nature of the phosphoproteins we had identified, appeared to me to have diverged from the cell cycle. Unfortunately, Mark Madsen had to leave Leicester but, before he left, he introduced me to Derek Raine, a physicist who wished to address biological problems and who has become a long-term collaborator. Brian Goodwin [86] and Mae-Wan Ho [87] from the *Open University* kindly came to Leicester to help us. From them, Derek and others, I learnt about some of the concepts in complexity studies such as autocatalytic sets, catalytic closure, and *edge of chaos*; in particular, there was the use of the (*N*, *k*) *model* to find a possible solution to the problem of how cells manage to generate reproducible phenotypes from an apparently hyper-astronomical number of combinations of expressed genes [88]; this has been a problem to which I have often returned (see below).

My interests in theory led to a long correspondence with Herb Kubitschek and Steve Cooper, who had very different views about whether the growth rate of individual bacteria was exponential or not [89,90]. I filed their letters in the same box, but there has been no spontaneous combustion as yet. Steve later took on the task of cleaning the *Augean Stables* of the literature on ‘synchronising’ eukaryotic cells, a Herculean task that has not been fully appreciated [91]. It did seem that even the fundamentals of the cell cycle field could be questioned, which resonated with my experience of *Psychology*. These questions included whether the bacterial cell cycle was best thought about in terms of phenotypic monotony (i.e., the average cell) or phenotypic diversity, whether chromosome segregation and cell division occurred via zonal growth or via a cytoskeleton, and whether regulation was principally by histidine kinases or, in the case of the cell cycle, by eukaryotic-like STY kinases.

Many of us in the field felt that it was time to shift the paradigm that bacteria had neither cytoskeletal structures nor STY kinases and had a cell cycle controlled by the dynamics of the envelope. However, our attempts to argue our case in one of the leading journals were unsuccessful [92]. This motivated me to write a ‘guide for editors’ and sent it to John Maddox at *Nature*. Maddox did not publish it because, in his words, ‘people might think he found it useful’, but he did invite me to write a review of the bacterial cell cycle. In response, my collaborators, Dave Sigee and Geoff Turnock, and I tried to write something original. We drew on the observations made in several labs that the candidates for the cytoskeleton often turned out to be enzymes (though we ourselves had failed to show structures based on DNA gyrase [93]) along with evidence for roles for calcium, phosphorylation, and membrane domains. We called this ‘cytoskeleton’ the ‘enzoskeleton’ in our review, which was not to the taste of *Nature*’s biology editors. It was, however, published elsewhere [94].

Naively, I had not realized that setting up a theoretical biology group was at variance with the overall research strategy for biology in Leicester. It was made clear to me that my time there was up. I therefore explored the possibility of working in some other field where my background in microbiology, psychology, and computers might be relevant. One of the most fundamental problems in science is the nature of subjective experience. I attended conferences on consciousness in Cambridge and in Tucson to see if I could interest anyone in the idea that bacteria might have subjective experiences or *qualia* [95,96]. I have revisited this idea recently in proposing that the operation of *competitive coherence* in bacteria generates patterns of activity that are inseparable from *qualia* and have illustrated this using the *Coco* program [97]. Fortunately, our ‘enzoskeleton’ paper had come to the attention of people in Rouen, France, who had just established an institute for multi-disciplinary science. The group in Rouen then invited me to join them.

## 5. Rouen, France 1996–Present

Our *Integrated Systems Institute* in the *University of Rouen* comprised several laboratories from across the disciplines. I first joined Janine Guespin’s laboratory of Microbiology to practice ‘integrative biology’ (as opposed, perhaps, to ‘disintegrative biology’?). This was a difficult time as my position was temporary and Janine and Camille Ripoll had to work hard to make it permanent. Janine’s interests included epistemology, which led to our attempts to define integrative biology [98], and epigenetics, which led to her collaboration with René Thomas (who himself had collaborated with Dick D’Ari [99]) and with others to use logical analysis [100]. Along with Chantal Monnier in Janine’s lab, I initiated a project with physical chemists (Jean-Marc Valleton, Stephane Alexandre, and Celine Fontaine) and biologists in Houston (Bill Margolin and Eugenia Mileykovskaya) to try to construct an in vitro division system based on membrane domains and the FtsZ protein using *Langmuir films*, *Atomic Force Microscopy*, and *Brewster Angle Microscopy* [101,102,103,104]. Another collaboration with physical chemists (Camille Ripoll, Rabah Boukherroub, and others) and biologists (Aaron Bensimon, Armelle Cabin-Flaman, Laurent Jannière, and others) entailed combining the techniques of *DNA combing* and *Secondary Ion Mass Spectrometry* (SIMS) so as to perform *C*ombing and *I*maging by *S*IMS or *CIS* [105,106]. This combination of techniques holds out the promise of revealing the rate of DNA replication on the scale of 1 kb. One of the many applications of *CIS* lies in investigating local variations in the rate of DNA replication; in particular, *CIS* could be used to test the idea that altering (or not altering) the rate of replication at specific places in the chromosome could affect the assembly of hyperstructures and thereby influence the phenotype [107]. Camille Ripoll and I were helped by Guillaume Legent, Anthony Delaune, Cecile Verrier, David Gibouin, and others in trying to develop a method to obtain the proteomes of individual cells using *Secondary Ion Mass Spectrometry* since this technique is so sensitive it can detect a single protein and localize it on the scale of tens of nanometers [108]; we envisaged constructing either a microarray of antibodies (isotopically distinct from their unlabeled targets) or an isotachophoretic separation microdevice (a microscale version of a stacking gel) [109]. Such approaches, which could reveal the extent of phenotypic diversity, led to a couple of patents.

Janine, Camille, and Michel Thellier in our *Integrated Systems Institute* were closely associated with the *Francophone Society for Theoretical Biology*, of which the President was Yves Bouligand. Yves had conducted pioneering work on liquid crystals in biological systems, which led to our hypothesis that daughter chromosomes with different cholesteric pitches would be immiscible, and that this immiscibility would be important in their separation [110]. Another pioneer in this area is Avi Minsky, with whom I have also collaborated; Avi had found that plasmids can also exist as liquid crystals [111]. One idea that came out of interactions with physicists and mathematicians like Gerard Gouesbet and Gerard Grehan in our institute was that reptation might help organize the cytoplasm into rivers in which polymers like RNA and DNA would flow; Yves and I talked about how this might lead to the chromosomes pirouetting, though we never took this as far as experiments and simulations. It does indeed now seem that reptation is important in biology [112,113,114].

My research in France benefited from the participation of the Rouen institute in the *Epigenomics Project*, which was set up in *Genopole* by François Képès and Paul Bourgine (and, later, Gilles Bernot), which brought together scientists from across the disciplines to work on problems in biology, and which ran for fourteen years. One of the topics was *hyperstructures*, tentatively defined as a large assembly of many molecules and macromolecules that serve a function [115,116,117]. The existence of a *hyperstructure* level of organization intermediate between the macromolecule and the bacterial cell offers one solution to the combinatorial problem raised by Stuart Kauffman because it reduces the number of elements required to determine the phenotype from thousands of macromolecules and genes to scores of hyperstructures. Hyperstructures are, in a sense, a natural extension of the *enzoskeleton* [94] and of *metabolite-induced metabolons*, a term coined by Milton Saier [118]. They appealed to my colleagues, Michel Thellier and Camille Ripoll, who had long been interested in macromolecular assemblies and the effects on catalysis of changes in concentration [119]. They dubbed one class of these assemblies *functioning-dependent structures*, which were forming because of their catalytic activity, an activity they possessed because they were forming [120].

One of the first steps in developing a subject is to create a taxonomy. We tried to do this in the case of hyperstructures, which represent a level of organization intermediate between macromolecules and the bacterial cell itself [115,121]. Paul Bourgine advised me to use a ‘generous Darwinian fog’ as it is important not to constrain a concept too tightly with a rigid definition at an early stage of its development. *Hyperstructure* is therefore something of a ‘catch-all’ term. Lois LeSceller and Patrick Amar modeled their dynamics using cellular automata [122,123] and investigated their possible contribution to metabolic efficiency [124], which many others have also carried out [125,126,127,128]. Subsequently, we have speculated about how anti-bacterial therapies might be developed using hybrid molecules or *hybolites* to cause the assembly of phenotypically incompatible hyperstructures [129,130].

Bacterial cells have evolved to survive harsh conditions and to grow in favorable ones: this requires a balancing act or *life on the scales of equilibria*, which is achieved via the dynamics of hyperstructures [131]. We have proposed that two classes of hyperstructures exist: equilibrium hyperstructures, which do not require a flow of energy to exist, and non-equilibrium hyperstructures, which do require this flow [115,116]; the former are required for survival and the latter for growth. To reconcile the apparently incompatible strategies required for survival and growth, we took the hyperstructure idea further by proposing that one of the functions of the cell cycle is to generate daughter cells with different phenotypes based on their inheritance of one or other of the classes of hyperstructures [132]. In following this trail to the *snark*, the cell might need to sense the quantity of equilibrium material and the intensity of the activity of non-equilibrium material and then integrate this information to trigger the cell cycle [131,133]. The question then is ‘how?’.

The generation of a reproducible range of coherent phenotypes could be achieved if the distribution of these classes of hyperstructures to the daughter cells were linked to the nature of the DNA strands. This would be because each daughter cell in receiving a different parental strand would also receive the different hyperstructures associated with this strand; we termed this *strand-specific segregation* [134]. Put differently, non-equilibrium hyperstructures would accompany one parental strand, while equilibrium hyperstructures would accompany the other. To back this up, Yoan Konto-Ghiorghi, Klara Kayser, Georgi Muskhelishvili, and I have found evidence in the literature consistent with the *Nucleoid-Associated Proteins* and topoisomerases assembling into hyperstructures that might exhibit strand-specific segregation [135,136]. In giving this importance to the strands, we were refining earlier ideas about the inevitability of differentiation arising from having two, essentially identical, chromosomes in the same cell [70]. Yannick Gangwe Nana in our group performed experiments in which he pulse-labeled bacteria with the stable isotopes ^15^N and ^13^C, and then, using *Secondary Ion Mass Spectrometry* [137,138,139], analyzed these bacteria [140]. The analysis of these incorporated isotopes revealed not only differences between individual bacteria that reflected major differences in growth rates (in what we assume was a population in steady-state growth) but also differences within individual bacteria that we interpret as corresponding to equilibrium and non-equilibrium hyperstructures. In the latter case, there was a significant difference between one half of the cell and the other half that we thought might give rise to the different phenotypes of the future daughter cells [140]. I was puzzled by the fact that this asymmetry between the cell halves *increased* with labeling time (up to three generations) and was slow to realize that this pattern, which echoed the results of the classic Meselson–Stahl experiment [141], could be the result of the combination of the strand-specific segregation of hyperstructures and the semi-conservative replication of DNA.

The realization that the generation of a coherent phenotypic diversity might even *be* the reason cells have adopted the semi-conservative method of DNA replication (rather than some other method) came to me in another ‘divine revelation’, albeit a belated one. Camille and I were able to explore this idea via simulation using a ‘chemostat’ containing a population of virtual bacteria that grew, segregated their DNA and associated hyperstructures semi-conservatively, and then divided in a chemostat [142]; this did indeed generate a population with a wide diversity of growth rates but, surprisingly, with little reduction in the overall growth rate (so at little cost in terms of selection on this criterion alone).

My hunt for the *snark* was guided by the hope that the control over the modern bacterial cell cycle would be rooted in the control over the growth, replication, and division of the first cells to have appeared. Insights into the former might therefore be gleaned from insights into the latter. Derek Raine and I continued to collaborate to explore various hypotheses about the origins of life, including those based on lipid domains [143,144]. In a think-tank organized in the context of the *Epigenomics Project*, a like-minded group of us proposed the *prebiotic ecology* (Derek Raine’s term), in which populations of mixed assemblies of different molecules evolved in an abiotic flux of creation and degradation, in which those molecules that interacted with one another via *molecular complementarity* [145,146] were preserved from degradation and therefore accumulated and underwent exchanges [143,147,148]. These *composomes* (for ‘compositional inheritance’ as simulated in [149]) were the putative ancestors of hyperstructures, and, in the *Accordion model* and related models, a dynamic interface between lipid domains in *composomes* (which Derek and I called ‘protocells’ at the time) catalyzed monomer to polymer reactions and became decorated with peptides and nucleotides that favored their own catalysis [144,150]. We further reasoned that, in our origins-of-life scenario, direct interactions between simple, universal molecules and ions (SUMIS), such as poly-(R)-3-hydroxybutyrate, polyphosphate, lipids, and calcium, would have played the central role in solving the fundamental problems confronting early and modern cells [151]; these problems include the generation of coherent phenotypes and the regulation of the cell cycle. More recently, I have collaborated with Jacques Demongeot (another President of the *Francophone Society for Theoretical Biology*) who, along with others, has proposed that a minimal RNA ring was selected in the origins of life that was particularly stable and that had one representative of each codon’s synonymy class [152,153]. Jacques and I first proposed that these rings might have generated assemblies of peptides that could have included ‘infinite’ proteins [152]. An intriguing problem is why life should have chosen a hereditary material that forms a double helix, which is hardly the ideal structure to facilitate the separation of its constituent strands needed for replication, segregation, and transcription. One possibility is that the eversion of small, double-stranded, polynucleotide circlets catalyzes the polymerization reactions that yield RNA, DNA, and peptides; this *Circlet hypothesis*, which also helps explain the triple code and the limitation to the twenty amino acids used by cells, is part of a larger *Ring World* hypothesis [154]. One of its predictions is that some of the DNA sequences encoding enzymes in modern cells have retained their original catalytic properties, such that a metabolic hyperstructure could contain both genes and enzymes catalyzing the same function.

Derek Raine visited us in Rouen in 1999 for a couple of weeks. On arriving, he told me that he had been reading Per Bak’s *How Nature Works* [155] and wanted to look for *self-organized criticality* in the changes in free energy (the delta G’s) of metabolic reactions. Although I guessed that this was a very interesting idea, I was dismayed as I had failed to appreciate Bak’s book and had other suggestions for Derek (moreover, I was uncertain about obtaining enough delta G’s). To get it out of his system, I gave him a *Nicholson* metabolic pathways chart and suggested that he look for the characteristics of *self-organized criticality* in considering the metabolites as nodes and their connections to other metabolites as links. The next day, he showed me his graph on which he had taken the connectivity of each metabolite and had then plotted the number of metabolites with the same connectivity; his graph had the power law and long tail characteristic of *self-organized criticality*. He then obtained a similar result from the bigger *Boehringer–Mannheim* chart [156]. Although we may have been the first to discover this, or at least to talk about it in public, I felt uneasy and wondered if it might be some artifact of the number system. This led Maurice Demarty and me to create a kind of artificial chemistry in which sequences of numbers acted as enzymes that catalyzed reactions between other sequences of numbers [157] (this was not as original as I had thought [158]); one conclusion was that different autocatalytic sets could destroy one another and that this could be prevented if they were segregated into different cells via the cell cycle, which offered a different view of the nature of the *snark*. Derek’s interest in connectivity continued first with Yohann Grondin and Michel Thellier in a thermodynamics approach to networks [159] and later in an investigation of the relationship between the overall connectivity of regulatory genes and their activity, as reflected in mRNA abundances [160].

My own interests in connectivity extended to its relationship with the cell cycle. The idea of the *initiation mass* has been entrenched in the thinking of cell cyclists for over fifty years [13,14,161,162,163,164,165,166,167], and a key protein that might sense when the cell has attained this mass has been identified as the DnaA protein [17,27]. But why should there be such a mass (if it exists) and why should it be one size rather than another? The existence of a cell depends on the patterns of connections between its constituents. This means that, if the cell were to keep on growing without dividing, the average connectivity between its constituents per unit mass would decrease and the cell would lose its identity. One solution would be for the growing cell to sense this decreasing connectivity and to trigger the processes that lead to cell division and thereby restore connectivity [132]. In this hunt for the *snark* in connectivity territory, the question then becomes as to how the cell senses decreasing connectivity; I therefore returned to hyperstructures as being on the right level of organization for me to speculate.

We had previously suggested that there might be significant differences in the pattern of connectivity within an equilibrium hyperstructure compared with a non-equilibrium hyperstructure, with the former having a few strong interactions between its constituents and the latter having many weak interactions between them [168]. In the *Dualism hypothesis*, the cell cycle is driven by the need to maintain the right ratio of non-equilibrium to equilibrium hyperstructures, such that the decrease in this ratio during growth triggers chromosome replication [133]. In thinking about macromolecules, hyperstructures, and the cell cycle, I found it hard to ignore water given that water constitutes around 70% of the mass of a cell and that 99% of its molecules are water. At one water conference, I met Frank Mayer, who had not only discovered cytoskeletal filaments of EF-Tu in *E. coli* [169] but also a possible relationship between membranes, water structure, and enzymic activity [170]. At another water conference, I met Jacques Benveniste, whose brave pursuit of a water *snark* had resulted in him vanishing scientifically. For many years after these conferences, I corresponded with Philippa Wiggins, who was an advocate of two-state water [171,172]. This led to the *Dualism hypothesis* invoking water structures in the dynamics of hyperstructures. *Dualism* also invoked ion condensation. I had been introduced to *Oosawa-Manning ion condensation* by Camille [173,174,175]. In this phenomenon, calcium and other counterions condense onto or decondense from linear polymers such as cytoskeletal filaments at a critical value of their charge density in what resembles a phase transition, and we proposed that the entire network of macromolecules in the cell (i.e., its hyperstructures) transduces and integrates information [175]. This was for me a case of Eliot’s ‘And the end of all our exploring will be to arrive where we started and know the place for the first time’. The abruptness of ion condensation and decondensation made it a good candidate as the physical mechanism responsible for the initiation of chromosome replication, with ion condensation on ribosomal hyperstructures and decondensation from the origin hyperstructure leading to the separation of strands at *oriC*; as mentioned above, *Dualism* has become *life on the scales of equilibria* where the balancing act performed by cells via ion condensation and the cell cycle is between survival-conferring equilibrium hyperstructures and growth-conferring non-equilibrium hyperstructures [131].

Over the last couple of decades, we have continued to develop the hyperstructure concept. In Leicester, we used mass spectrometry to study the lipid composition of bacterial membranes (where I made a silly error in labeling the graph [176]). In Rouen, we continued this analysis with Delphine Oursel, Corinne Loutelier-Bourhis, and Catherine Lange in *Chemistry* and Nicole Orange in *Microbiology* [177,178], the ultimate objective having been to determine the lipid preferences of proteins by overproducing them so as to obtain a cartography of membrane hyperstructures (this was inspired by results obtained by others [179,180]). To understand such hyperstructures better, I have collaborated with Kouji Matsumoto [181,182], whose group found that the enzyme responsible for cardiolipin synthesis in *B. subtilis* is located in the cardiolipin-rich septal membrane [183]. Such understanding was greatly increased by Dennis Bray’s insight that the sensitivity of a cell to a chemoattractant could be regulated by the size of the hyperstructure of intercommunicating receptors to that attractant [184]. This was one reason Armelle Cabin-Flaman, Camille Ripoll, Milton Saier Jnr, and I speculated that the *transertion* of the receptors had to be separated from the hyperstructures in which they function [185]. We also attempted to relate hyperstructures to pathogenicity by showing how interactions between hyperstructures, like the T3SS injectosome and the RNA degradosome, might influence virulence potential based upon the physical locations of hyperstructures within the pathogen [186]. This hypothesis had its origins in a student presentation of a paper that illustrates the importance of performing controls that appear superfluous: “unexpectedly, a catalytically inactive PNPase restored normal T3SS activity to these Δ*pnp* strains; this was especially surprising given the fact that an active PNPase is absolutely required to restore the cold-growth defect of the *Yersinia* Δ*pnp* strains” [187].

In our membrane-based pursuit of the *snark*, Eugenia Mileykovskaya, Conrad Woldringh, and I exploited the possibility that domains in the plane of the membrane could invaginate to form tubes [188]; we proposed that these tubes are the substrate for FtsZ, which by changing its polymeric conformation converts them into the invaginating fold responsible for division [189]. I was particularly happy with this combination of two physical processes to solve—rightly or wrongly—a biological problem. Conrad also had a paper with Itzhak Fishov on the relationship between membrane and DNA that shows changes to the membrane occurring at or close to the time of initiation of replication [190]. Michi Kohiyama, John Herrick, and I have used this result to argue that a single event triggers both DNA replication and cell division, which simplifies the hunt for the *snark* [191]. In this paper with Michi and John, we again invoke theconnections between central carbon metabolism and replication that have been uncovered by Laurent Jannière and his collaborators [192,193], which could take the form of local changes in the rate of replication, leading to differential gene expression [107]. This is all part of a rethinking of the cell cycle in which its primary function becomes, producing two phenotypically different daughter cells, leading to the phenotypically diverse population that allows growth or survival in diverse environments.

In continuing to think about water, central carbon metabolism, and the cell cycle, I recalled some surprising findings in papers that I had filed away because I had no explanation for them. These papers reported that a high proportion of intracellular water comes from metabolism and, principally, glycolysis [194,195,196,197] (but see [198]); moreover, the reactants and products in metabolic reactions may bind water or release bound water, which may be particularly important in the case of ADP and ATP [199] where the high level of intracellular ATP has lacked a convincing explanation [200,201]. I interpreted this to mean that the cell had the possibility of varying its water content, which would mean altering the dynamics of hyperstructures. In the *water-clock* hypothesis, I have proposed that two sorts of water—free water and hydration water—are central to the timing of the cell cycle [202]. In essence, water becomes limiting as the cell grows, which causes a set of phase transitions that create and then change the hyperstructures responsible for the initiation of chromosome replication; this is *life on the edge of phase transitions* [202]. Recent evidence does indeed show that limiting water can cause a phase transition that leads to the assembly of condensates, a class of hyperstructures [203,204]. The advantages of this *water-clock* hypothesis include coordinating catabolism (which increases free water) and anabolism (which reduces free water), coupling mass increase to DNA replication, providing both a quantity and an intensity sensing mechanism, and rooting the cell cycle of modern cells in the *Ring World* scenario of the origins of life [202]. Many years ago, Michel Thellier told me that if there really were an organizational level of hyperstructures, they would have their own language: in the *water-clock*, this language is water.

## 6. Miscellaneous Research

As a theorist in an integrated systems environment, I have dabbled in several fields. One of these fields is plant biology and, in particular, the study of how plants remember external stimuli. This was conducted by Michel and Marie-Odile Desbiez [205] and continued by Camille and Marie-Claire Verdus [206]. I made a small contribution to it with the *Theatre Management Model*, in which we tried to interpret plant memory in terms of *competitive coherence* [207]. We also resumed our collaboration with Glenn White to explore the effects of exposure to low-intensity, non-ionizing radiation in the GHz range in a sensitive meristem system [208], effects which meant that Marc Tafforeau was able to implicate calcium and protein phosphorylation [209].

Thanks to the *Epigenomics Project*, I have collaborated with Judit Ovadi and her group several times. These collaborations began with our proposing that the eukaryotic cytoskeleton senses and integrates the general metabolic activity of the cell by changing its dynamics in response to it binding many different enzymes according to their metabolic activity [210,211]. We extended this thinking into the COVID-19 field, where we proposed anti-viral strategies based on the cytoskeleton and on defective-interfering particles [84] (Maria Kalamvoki and I were to develop the latter strategy further [85]). My collaboration with Judit has also been in the field of neurodegeneration, where she and her lab have identified *Tubulin Polymerization-Promoting Protein* as a major pathogenic partner of alpha-synuclein, which has implications for therapies [212,213]; we have given this a theoretical underpinning in the *Sherpa hypothesis*, where we propose that these intrinsically disordered proteins be considered as *Phenotype-Preserving Disordered Proteins* that protect cell phenotypes from perturbations [214]. Intrinsically disordered proteins and proteins with intrinsically disordered regions [215] are important in the hypotheses we have proposed with respect to water availability [202] and the replication of DNA (as in the cases of single-stranded binding protein and DnaA) [191]. In these hypotheses, the initiation hyperstructure acts in a way analogous to the integrative sensing we have proposed for the eukaryotic cytoskeleton [210]. Another benefit of the *Epigenomics Project* was the opportunity to interact with its guest speakers such as Marc-Emmanuel Dumas [216] and Andrew Gewirtz, which led to our proposal that the gut microbiome actually helps determine what we choose to eat [217].

Over the years, I have had the good fortune to pursue quarry other than the *snark* itself. One of these is how operons are made, a quarry that Annabelle Merieau and I pursued in the context of the *Elie Wollman Prize* with the idea that plasmids could act as *scribbling pads*, which could be used by cells to rearrange the positions of genes on the chromosome [218]. Another of our quarries has been the reason why organelles like mitochondria have DNA when most of their proteins are imported, which was considered to be unclear at that time (and may still be [219]). Given the relationship between bacteria and organelles, we looked for an explanation in terms of hyperstructures, and with Mirella Trinei, Marie-Beurton-Aimar, and Jean-Pierre Vannier (I was attached to Jean-Pierre’s lab at the time, which was working on *acute lymphoblastic leukemia* in children), we proposed that this is because transcription–translation hyperstructures are needed to structure these organelles [220]. More recently, I have worked with Alexei Sharov to introduce the concepts of bacterial hyperstructures and *competitive coherence* into biosemiotics [221].

There have been quarries in synthetic biology too. In the case of the *Mimic Chain Reaction*, we have proposed a generic method, analogous to the *Polymerase Chain Reaction*, for amplifying proteins or other molecules based on bacterial autoinducer systems [222], on which Yannick Gangwe Nana has worked extensively, whilst in the case of obtaining catalysts to any reaction, we have proposed that switchable enzymes or *swenzymes* could be constructed from DNA aptamers to bind substrates and catalyze reactions when exposed to external energy in the form of a specific frequency of low-intensity, non-ionizing electromagnetic or acoustic radiation [223]. In the case of biological computing, we have proposed using populations of bacteria to perform *bactoputing*, which exploits their natural behavior based on hyperstructure dynamics and the cell cycle to solve, in principle, a variety of problems, including the combinatorially challenging *NP-hard* ones (i.e., problems that cannot be solved in polynomial time but have answers that can be verified in polynomial time) [224]. An argument can be made that tribal species like our own, whose capacity for thinking has evolved on the anvil of struggles with other tribes, are doomed to destroy themselves once they acquire a certain level of technology. We have therefore proposed that a final task for synthetic biology may be to leave a message for future species by writing it into the genome of bacteria, assuming that it is not already there [225,226].

## 7. Future Projects

In mentioning the following, my intention is not to try to stake a claim to them. Far from it. Anyone is welcome to them (for what they’re worth). In the ideal scientific world, there would be no secrets. To quote Richard Ashcroft, “I don’t wanna know your secrets, they lie heavy on my head”.

The study of hyperstructures would benefit greatly from the application to bacteria of toponomics, which can localize up to a hundred different macromolecules on the nm scale [227]. The activity of these hyperstructures might then be revealed by combining toponomics with pulse-labeling with a stable isotope followed by *Secondary Ion Mass Spectrometry* [140]. In the *Dance floor* model, one mechanism responsible for the assembly or disassembly of hyperstructures during the cell cycle would be on the basis of a water-dependent phase separation of their constituents by their mutually exclusive, conformational oscillations. Another mechanism that might affect the assembly of hyperstructures and the communication between them would be if the level of hyperstructures were to exhibit the coherent dipole oscillations proposed to act at the level of the cell [74]. Another aspect of hyperstructures came from my visit to Abdallah Zemirline and Pascal Ballet (also members of the *Epigenomics Project*), which led to the idea that travelling phase transitions or *phoscillons* could occur—or be made to occur—in the plane of biological and artificial membranes, and even in 3D [228]; this is not a million miles from Dennis Bray’s ‘conformational spreading’ [229].

DNA is likely to be central to my future work or, with luck, to that of my collaborators. It would be fun to return to the Z-DNA story and to see whether a propensity to form Z-DNA is correlated with the *oriK* sites where constitutive stable replication can be initiated [230]. It would also be fun to explore the possibility that DNA supercoiling naturally produces plectonemes of a relatively constant length if there is a negative constraint proportional to this length (an idea that has its origins in my childhood experiences with tangled, supercoiled, fishing lines). It should also prove possible to use the *water-clock* hypothesis to re-interpret the wealth of data about the initiation of DNA replication in both prokaryotes and eukaryotes. One way to do this would be to adapt the program we used to study the relationship between strand segregation, hyperstructures, and growth rates [142]; this would entail introducing non-equilibrium and equilibrium hyperstructures coupled with anabolic and catabolic functions (consuming and producing free water, respectively), metabolic reactions such as the hydrolysis of ATP (and accompanying decreases in water availability), and an initiation-dependent sensing of water. Initiation at the origin of replication is followed by inactivation of the newly replicated origins via sequestration by the SeqA protein [231]; another, more fundamental, way to prevent premature reinitiation would be for the phase separation of the two parental strands to separate the factors needed for initiation by exploiting semi-conservative replication. My longstanding conviction that the cell cycle is essentially a process of differentiation [68] has been given a new lease of life in the idea that, if decreasing water availability was to trigger a set of phase transitions responsible for triggering the initiation of replication, the same transitions could, if partial, also separate macromolecules into equilibrium and non-equilibrium hyperstructures and hence also confer phenotypic diversity on future daughter cells. Exploring this idea would benefit from knowing how many water molecules bind to each of the cell’s major constituents, which would allow for the ‘water accountancy’ needed.

Again, on the programming front, my search for the *snark* will hopefully bring together *competitive coherence* and the cell cycle by simulating a ‘chemostat’ containing a population of virtual bacteria growing or surviving according to how well they learn to respond to their environment. Finally, I have long been impressed by the beauty of tensegrity and its applications to cell biology, biotechnology, and medicine [232]; it would be fun to try to extend these applications to the worlds of taxation and stock exchange dynamics, as we have tried to do with the *Coco* program [233].

The concepts we have adopted or developed in the context of the origins of life (which include structures with different time scales, the generation of variability, catalysis via the hereditary material, fission–fusion, growth and survival, and *competitive coherence*) might be tested to the limit in considering whether the Sun contains life or indeed is itself is alive; this speculation has a long history [234,235].

## 8. Discussion

**Figure 1:**
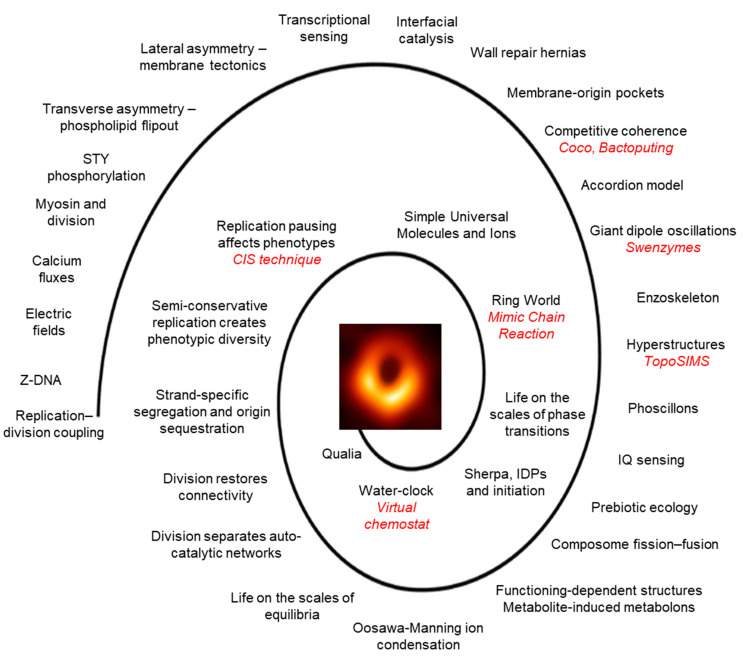
Approaches to the cellular *snark*: The spiral represents the route I have taken to the *snark*, which is itself represented as a supermassive black hole in the center of the M87 galaxy. Techniques that may be useful in exploring a particular approach are in red italics. The image of the black hole was captured by FORS2 on ESO’s Very Large Telescope (https://science.nasa.gov/resource/first-image-of-a-black-hole/). accessed on 21 September 2024.

During the 42 years I have been working on the bacterial cell cycle (Figure 1), I have the impression that it has become increasingly attractive to physicists and physical chemists. I also have the impression that the goal of physics is to find elegant, quantitative solutions to problems that can be simplified. But is this the right sort of hunt to find the *snark*? It seems to me that biology is different from physics in having a vast, non-exclusive number of potential solutions to its problems. This is because of the vastness of *solution space* that has been created by many millions of species [236] under selection over billions of years in hyper-astronomical combinations of environmental conditions. In the case of the cell cycle, I have argued that our hunt should be for a bacterial *snark* because bacteria were here first, made our world, make up much of the biomass, determine our behaviour, and constitute some of biology’s most tractable and best understood model systems [97]. Not everyone is of this opinion.

To try to increase the perceived importance of the bacterial cell cycle, we recently created the *Charles E. Helmstetter Prize for Groundbreaking Research in Bacterial Cell Cycle Physiology* [237], named in honour of the person who, with Steve Cooper, laid the foundations of the modern bacterial cell cycle [238]. Deciding on the criteria for awarding such a prize depends in part on what one thinks science itself is (one might ask the question whether science is more about getting it wrong than getting it right [239]). This kind of questioning has been my experience with physicists, who often interrupt my description of my research interest by asking ‘but what is the question?’. In other words, what is the nature of the *snark*? (For what it’s worth, the cell cycle may be the solution to the problem of mapping the environmental diversity of a species – corresponding to the environments in which it can grow or survive – onto its phenotypic diversity).

My experience as reported above, is that the nature of the *snark* changes as a function of the nature of the hunt. My hunt began with molecular genetics and biochemistry with an image of the *snark* as a system regulating the cell cycle of a cell comprising genes and proteins. This hunt has been transformed into one based on physical chemistry and physics whilst the *snark* itself has metamorphosed into a system regulating something unknown. This unknown is the cell. Asking ‘what is a cell?’, leads me to the answer that the cell is more than an autocatalytic system, a dissipative system, a tensegrity system, a system on the edge of chaos (or on the edge of phase transitions or on the scales of equilibrium and non-equilibrium hyperstructures), a neural net, a multi-level competitive coherence system, and a generator of *qualia*: rather, the cell may be all of these descriptions – and – then some [240]. In short, the cell is the creator and the creation of an extraordinarily high density of different organizing processes that interact with one another: the cell is both poet and poem. And only when we fully understand this poem will we be able fully understand the cell cycle and catch the *snark*:

“For the Snark’s a peculiar creature, that won’t

Be caught in a commonplace way.

Do all that you know, and try all that you don’t:

Not a chance must be wasted to-day!”

## Data Availability

No new data were created or analyzed in this study. Data sharing is not applicable to this article.
